# Invasive* Candida* Infection after Upper Gastrointestinal Tract Surgery for Gastric Cancer

**DOI:** 10.1155/2017/6058567

**Published:** 2017-11-06

**Authors:** Evgeni Brotfain, Gilbert Sebbag, Michael Friger, Boris Kirshtein, Abraham Borer, Leonid Koyfman, Dmitry Frank, Yoav Bichovsky, Jochanan G. Peiser, Moti Klein

**Affiliations:** ^1^Department of Anesthesiology and Critical Care, General Intensive Care Unit, Soroka Medical Center, Ben-Gurion University of the Negev, Beer Sheva, Israel; ^2^Department of General Surgery B, Soroka Medical Center, Ben-Gurion University of the Negev, Beer Sheva, Israel; ^3^Department of Public Health, Faculty of Health Sciences, Ben-Gurion University of the Negev, Beer Sheva, Israel; ^4^Department of General Surgery A, Soroka Medical Center, Ben-Gurion University of the Negev, Beer Sheva, Israel; ^5^Department of Infectious Disease, Soroka Medical Center, Ben-Gurion University of the Negev, Beer Sheva, Israel; ^6^Department of Medical Management, Soroka Medical Center, Ben-Gurion University of the Negev, Beer Sheva, Israel

## Abstract

Upper gastrointestinal tract (GIT) surgical procedures are more likely to cause nosocomial* Candida peritonitis* than lower GIT procedures and they thus constitute an independent risk factor for mortality. Because of the severity of postsurgical fungal infections complications, intensivists and surgeons need to be extremely aware of their clinical importance in critically ill postsurgical intensive care unit (ICU) patients. We analyzed the clinical and microbiological data of 149 oncologic patients who were hospitalized in the ICU at Soroka Medical Center between January 2010 and January 2015 after undergoing upper GIT surgery for gastric cancer. Invasive fungal infections related to secondary peritonitis following oncologic upper GIT surgery had a higher mortality rate than patients with nonfungal postoperative infectious complications. The presence of gastroesophageal junction leakage and advanced age were found to be independent risk factors for invasive fungal infection after oncologic upper GIT surgery.

## 1. Introduction

Gastrointestinal tract (GIT) surgery is a major risk factor for secondary peritonitis [1]. Surgical intervention causes this complication by altering the physiologic flora of the GIT and by directly damaging the natural barriers of infection [2]. In the wake of surgical intervention, the GIT is most often colonized by Gram-negative invasive microorganisms. However, under certain postsurgical conditions* Candida* fungi are liable to colonize the peritoneal cavity and cause infection [3]. It is known that upper GIT surgical procedures are more likely to cause nosocomial* Candida peritonitis* than lower GIT procedures [1, 4–6] and consequently upper GIT surgery constitutes an independent risk factor for mortality [4]. Underlying comorbidities, such as immunosuppression, cancer, the frequently malnourished state of critical care patients, administration of total parenteral nutrition (TPN), and use of intravenous catheters, are additional factors that tend to increase the frequency of* Candida* colonization and peritonitis in oncologic surgical patients undergoing upper GIT surgery [7, 8]. All the above factors when present in oncologic surgical patients undergoing upper GIT surgery significantly increase their risk for developing intra-abdominal* Candida* infections. Importantly, the overall mortality rate is much higher in surgical critically ill patients with intra-abdominal* Candida* infections than in those with purely bacterial infections [9, 10]. Furthermore, postsurgical patients who are critically ill with fungal or nonfungal secondary generalized peritonitis often require repeated laparotomies, which in turn are associated with a high incidence of yeast superinfection in the peritoneal fluid [4, 11]. Because of its severity, intensivists and surgeons need to be extremely aware of the clinical importance of fungal superinfection in critically ill postsurgical intensive care unit (ICU) patients and to pay scrupulous attention to the factors that predispose to these infections.

In the present study, we reviewed and analyzed the clinical and microbiological data of oncologic patients who were hospitalized in the General Intensive Care Unit (GICU) at Soroka Medical Center between January 2010 and January 2015 after having undergone upper GIT surgery for gastric cancer.

## 2. Patients and Methods

Soroka Medical Center is a 1000-bed tertiary care university teaching hospital located in the city of Beer Sheva in Israel's southern Negev region. We retrospectively collected the clinical and laboratory data on all patients who underwent oncologic upper GI surgery for gastric cancer and who were hospitalized postoperatively in the GICU at the Soroka Medical Center between January 2010 and June 2015. All the clinical data were extracted from the OFEK™ Registry Information System, the Operating Room Registry System, and the Metavision ICU Registry Information System. The study was reviewed and approved by the Human Research and Ethics Committee at Soroka Medical Center (RN 0334-15-SOR).

### 2.1. Inclusion Criteria

All patients aged ≥ 18 who underwent any type of oncologic upper GIT surgery for gastric cancer between January 2010 and January 2015 and who were hospitalized postoperatively in the GICU at Soroka Medical Center were eligible for inclusion in the study.

### 2.2. Exclusion Criteria

The following patients were excluded from the study: patients who were immunosuppressed (including those who had undergone preoperative chemo- and/or radiotherapy); patients with chronic and/or recurrent skin or mucosal fungal infections, such as intertrigo/oral candidosis, who were treated with antifungal therapy one month prior to hospital admission or who were known carriers of a fungal infection; and patients who had been hospitalized for more than one month prior to the upper GIT surgical procedure.

### 2.3. Variables and Measures

We recorded the following parameters: demographic data; the presence or absence of comorbid conditions; the patients' chronic medications; the type of primary surgery undergone by the patients as well as information regarding any reoperations; data on interventional procedures performed postoperatively; and the results of relevant imaging studies. Information regarding laboratory and microbiological studies was also recorded. Clinical data recorded included the patients' diagnoses on admission, the Acute Physiology and Chronic Health Evaluation-II (APACHE II) score, and the Therapeutic Intervention Scoring System (TISS) score. Other recorded parameters included the rate of success in weaning the patients from mechanical ventilation (the number of ventilator-free days); the therapeutic management of the patients; their nutritional state during their ICU stay; the development of infectious complications; and the intra-ICU and intrahospital mortality rates among the study patients.

### 2.4. Microbiology

The microbiological data included the results of blood, peritoneal fluid, and pleural fluid cultures sampled during the patients' hospital and ICU admissions. Intra-abdominal infection (peritonitis), bacteremia (noncentral line-associated blood stream infection {BSI}), and empyema were diagnosed according to the criteria specified in the international surveillance guidelines of the Centers for Disease Control [12]. An* invasive fungal infection* was defined as a new event of fungemia, fungal peritonitis, or fungal empyema after oncologic upper GIT surgery.

### 2.5. Definitions

The severity of illness and the presence or absence of multiorgan failure were evaluated using the patients' APACHE II and TISS scores as recorded within 24 hours of ICU admission.

### 2.6. Study Groups

The patients were divided into two groups. Group 1 comprised oncologic surgical patients who underwent elective upper GIT surgery for gastric cancer and had an uneventful postoperative course. Group 2 comprised oncologic surgical patients who underwent elective upper GIT surgery for gastric cancer and developed new postoperative intra-abdominal infectious complications.

### 2.7. Statistical Analysis

Data analysis was performed with SPSS (version 18.0 or higher). Data collected in this study was summarized using frequency tables, summary statistics, confidence intervals, and *p* values as appropriate. Continuous variables were compared by *t*-tests or analyses of variance. For continuous variables with nonnormal distribution, comparisons were evaluated for significance using the Wilcoxon rank-sum test. For categorical variables, the 95% confidence interval was analyzed using binomial distribution. For continuous variables, the 95% confidence interval was calculated using means and standard errors derived from Student's *t*-test statistical method.

## 3. Results

The clinical and laboratory data of 149 patients who underwent oncologic upper GIT surgery for gastric cancer were analyzed. Forty-nine (33%) of the patients developed secondary peritonitis and were hospitalized in our GICU during the study period (Group 2). The remaining one hundred patients (67%) had an uneventful postoperative course (Group 1). The patients' demographic data, their past medical history, and their clinical and nutritional parameters are presented in Table 1. The patients in Group 2 were significantly older than those in Group 1 (*p* value 0.002, Table 1). The two groups were similar for gender, weight, and type of upper GIT surgery. Underlying diabetes (type II) was more frequent among the Group 2 patients. In contrast, Group 1 included more patients with arterial hypertensive disease (Table 1). There was a significant difference in the frequency of chronic statin therapy between the two groups (Table 1). TPN after upper GIT surgery was initiated more often in Group 2 patients than in Group 1 patients (73.5% versus 27%, *p* < 0.001, Table 1). The duration of the patients' admissions, both in the ICU and in the hospital, was significantly longer in the Group 2 patients compared to the Group 1 patients (*p* < 0.001, Table 1).

Table 2 shows demographic data, postoperative infectious complications, and clinical outcome data of Group 2 patients only. All 49 patients in Group 2 had documented intra-abdominal infections (peritonitis). Ten of the 49 patients (20.4%) also had invasive fungal* (Candida albicans)* infection (*Candida peritonitis*, *n* = 5; candidemia, *n* = 3;* Candida empyema*, *n* = 2) on admission to the ICU (Table 2). Importantly, all three cases of candidemia were noncentral line-associated blood stream infection. There was no difference in the demographic data, type of surgery, and past medical history between the patients with and without fungal infections. A large proportion of the patients with nonfungal infections were receiving chronic therapy with ACE inhibitors (Table 2).

Patients with nonfungal and invasive fungal infectious complications had similar APACHE and TISS scores within 24 hours of ICU admission and similar lengths of ICU and hospital admissions (Table 2). Patients with invasive fungal infection had a higher incidence of intraperitoneal leak documented during surgery compared to patients with nonfungal infections (90% versus 69%, *p* value 0.01) (Table 2).

In the patients with invasive fungal infection, leakage was more frequently found at the gastroesophageal junction area compared to the patients with nonfungal infections (80% versus 34%, *p* < 0.02, Table 2). In contrast, the patients in the nonfungal subgroup had a higher frequency of leak at the gastrointestinal anastomosis and in the small bowel area (30–34% versus 10%, *p* 0.01 and 0.02, resp., Table 2). Also, there was a higher frequency of intra-abdominal abscesses and pleural effusions in the patients with invasive fungal complications as compared to those with nonfungal infections (*p* value 0.006 and 0.04, resp., Table 2).

The ICU mortality rate was much higher in patients with invasive fungal infectious complications compared to those without fungal infection (50% versus 15%, *p* value 0.03, Table 2).

Microbiological data of the Group 2 patients (Table 3) showed similar culture growth of* Streptococcus constellatus* and coagulase negative* Staphylococcus *spp. in the abdominal fluid, the pleural fluid, and the blood in both the nonfungal and the fungal subgroups. In contrast, there was a higher frequency of positive cultures of Gram-negative flora* (Escherichia coli, Pseudomonas *spp.,* and Klebsiella *spp.) in the abdominal fluid, the pleural fluid, and the blood in patients with nonfungal infectious complications (see Table 3) compared to those with fungal infections.

Patients with invasive fungal infections had a higher creatinine level on admission to the ICU than those with nonfungal infections (1.6 ± 0.2 versus 0.94 ± 0.58, *p* value 0.01). Other laboratory data did not differ between the fungal and the nonfungal subgroups (Table 3).

No difference was found between the fungal and the nonfungal subgroups in regard to the following therapeutic measures that were implemented while the patients were in the ICU: CT-guided drainage of pleural effusions and abdominal abscesses; nutritional care; administration of steroids; vasopressor therapy; and additional surgical interventions (Table 4).

Table 4 shows the results of multivariate logistic regression analysis of postoperative peritonitis after oncologic upper GIT surgery. Advanced age, underlying diabetes mellitus, and postoperative TPN treatment were found to be independent risk factors for postoperative secondary peritonitis in patients who underwent oncologic upper GIT surgery (Table 4).

Further multivariate analysis of postoperative invasive fungal infections in the wake of oncologic upper GIT surgery is shown in Table 5. Gastroesophageal junction leak and advanced age were found to be independent predictors for invasive fungal infections after oncologic upper GIT surgery (Table 5).

## 4. Discussion

Postoperative infectious complications following oncologic gastric surgery are known to be associated with a significant decrease in 5-year overall and relapse-free survival (66% and 64%, resp., in patients with infectious complications versus 87% and 85%, resp., in an uncomplicated population group) [13]. Advanced age, male gender, underlying cirrhosis, prolonged operative time, suturing or anastomosis of the small bowel, and total gastrectomy were found to be independent risk factors for postoperative infectious complications in patients undergoing upper GIT oncologic surgery [13, 14].

In our study, we retrospectively analyzed 149 cases of oncologic surgical patients who underwent upper GIT surgery for gastric cancer. The postoperative course of 49 patients (Group 2) was complicated by secondary peritonitis with an overall ICU mortality rate of 22% (11 patients). Advanced age, underlying diabetes mellitus, and postoperative parenteral nutrition were found to be independent risk factors for postoperative secondary peritonitis in these patients. In our study, ten (20%) of the Group 2 patients had the following invasive fungal (*Candida*) infections: candidemia,* Candida peritonitis,* and* Candida empyema*.

Over the last two decades several studies have described fungal complications after upper GIT surgery. Candidemia was reported in 10–20% of patients with nosocomial or complicated secondary and tertiary peritonitis [4].* Candida peritonitis* is associated with a markedly raised mortality rate which can reach as high as 60–70% [4, 5, 15], reinforcing the contention that* Candida* is an independent risk factor for mortality in peritonitis. Some authors [4, 5, 15, 16] have demonstrated almost a double mortality rate (48% versus 28%) in critically ill surgical patients with nosocomial fungal peritonitis compared to those without fungal superinfection [4].

Surgery itself is a major risk factor for* Candida peritonitis* [17–19]. Other risk factors for fungal infections after abdominal surgery were found to be recurrent gastrointestinal perforation, previous treatment with broad-spectrum antibiotics, parenteral nutrition, and central venous catheter insertions. The frequency of invasive fungal infections in the oncologic population continues to increase due to impaired host defenses resulting from underlying disease and/or immunosuppressive therapy [20]; however, the precise incidence of* Candida* infection after oncologic upper GI surgical procedures remains indeterminate.

In our study, in multivariate analysis, advanced age and the presence of a gastroesophageal junction leak were identified as independent risk factors for invasive fungal infectious complications after oncologic upper GIT surgery. In fact, the frequent occurrence of leakage at a high GIT location in our patients supports a primary source of* Candida* in the oral cavity. In previous studies,* Candida* colonization was isolated in 41% of upper GIT sites [6, 17]. Several studies [1, 4, 21] have demonstrated that the presence of yeast isolates in the oral cavity is about 35% in patients aged 56 to 70 but is much more frequent (up to 74%) in patients aged 71–92 years. It is not surprising, therefore, that up to 30–40% of patients with secondary peritonitis are liable to develop* Candida peritonitis* or intra-abdominal abscesses [4, 11]. Importantly, 90% of our patients with invasive fungal infections had a documented postoperative leak as well as a higher frequency of intra-abdominal abscesses and pleural effusions than those without fungal superinfection.

Of note, Edwards Jr. et al. [7] demonstrated that critical illness, parenteral nutrition, and corticosteroid therapy are also independent risk factors for fungal infection in the ICU. However, in our study, no differences in severity of critical illness, postoperative therapeutic management, and type of nutrition were demonstrated between the nonfungal and the invasive fungal populations.

Several previously published data have demonstrated an increased mortality rate in patients with dual infections with* Candida albicans* and* E. coli* [22, 23]. Furthermore, Sawyer et al., investigating the role of* Candida albicans* in the pathogenesis of mixed fungal and bacterial infections, found a synergistic effect on mortality rates in patients with* E. coli *and* B. fragilis* who suffered from simultaneous fungal superinfections [2]. In the present study, microbiological analysis of the peritoneal fluid, blood, and pleural effusions of the patients with invasive fungal infections showed a high frequency of concomitant* Streptococcus constellatus *and coagulase negative* Staphylococcus *spp. cultures (Table 3). In contrast, the frequency of* E. coli* positive cultures was similar (20–25%) in the peritoneal fluid of patients with nonfungal peritonitis and in that of the patients with concomitant invasive fungal infections and mixed abdominal flora (Table 3).

Our study has several limitations, the most important of which is its retrospective design. Another limitation was our inability to make an appropriate selection of the patients and to take into account the administration of antifungal therapy. The significance of our results for the long-term outcomes of our study patients is unclear because the study did not incorporate follow-up of these patients after discharge from the hospital.

## 5. Conclusion

In conclusion, secondary peritonitis has emerged as a significant postoperative infectious complication after upper GIT surgery for gastric cancer. In the present study, underlying diabetes mellitus, advanced age, and postoperative parenteral nutrition were independent risk factors for the development of peritonitis after oncologic upper GIT surgery. In addition, our study demonstrated that surgical patients who developed invasive fungal infections related to secondary peritonitis had a higher mortality rate than patients with nonfungal postoperative infectious complications. The presence of gastroesophageal junction leakage and advanced age were found to be independent risk factors for invasive fungal infection after oncologic upper GI surgery.

## Figures and Tables

**Table 1:** Patient demographics, underlying conditions, nutritional data, length of ICU, and hospital stay (Group 1: no infectious complications; Group 2: patients who developed documented intra-abdominal infection).

	Group 1 (*n* = 100)	Group 2 (*n* = 49)	*p* value^*∗*^
Age, years (mean ± SD)	62.67 ± 9.1	72.38 ± 14.2^*∗*^	0.002
Weight, Kg (mean ± SD)	72.78 ± 12.75	72.85 ± 13.7	0.7
Gender (male)	48/100 (48%)	24/49 (49%)	0.5
Type of upper GIT^a^ surgery			
Total gastrectomy	56/100 (56%)	26/49 (53.1%)	0.8
Partial gastrectomy	44/100 (44%)	23/49 (46.9%)	0.9
Underlying condition (%)			
Without chronic disease	36/100 (36%)	15/49 (30.6%)	0.6
Diabetes mellitus	10/100 (10%)	18/49 (36.7%)^*∗*^	0.04
CIHD^b^	31/100 (31%)	14/49 (35.8%)	0.5
Hypertension	23/100 (23%)	1/49 (2.04%)^*∗*^	<0.001
Chronic therapy (%)			
Without chronic therapy	36/100 (36%)	15/49 (30.6%)	0.46
Statins	20/100 (20%)	16/49 (32.6%)	0.04
ACE^c^	44/100 (44%)	18/49 (36.7%)	0.29
TPN (*n*, %)^d^	27/100 (27%)	36/49 (73.5%)	<0.001
Length of stay (day ± SD)			
ICU length of stay^e^ (day, mean ± SD)	1.25 ± 0.67	12.04 ± 1.49	<0.001
Hospital length of stay (day, mean ± SD)	10.84 ± 4.8	32.2 ± 2.4	<0.001

^*∗*^ *p* < 0.05 is considered to be significant; ^a^GIT: gastrointestinal tract; ^b^CIHD: chronic ischemic heart disease; ^c^ACE: angiotensin-converting enzyme. ^d^Percent total parenteral nutrition after oncologic upper GIT surgery. ^e^Some of the patients from Group 1 were also hospitalized in the GICU for a postoperative observation period.

**Table 2:** Demographic data, postoperative infectious complications, and clinical outcomes endpoints of Group 2 patients divided into nonfungal and invasive fungal subgroups (*n*, %, mean ± SD).

	Nonfungal (*n* = 39)	Invasive fungal^a^ (*n* = 10)	*p* value^*∗*^
Age, years (mean ± SD)	72.25 ± 14.29	72.9 ± 14.9	0.15
Weight, Kg (mean ± SD)	72.9 ± 12.9	72.6 ± 13.57	0.9
Gender (male)	21/39 (53.8%)	3/10 (30%)	0.4
Type of upper GIT^b^ surgery			
Total gastrectomy	22/39 (56.4)	4/10 (40%)	0.61
Partial gastrectomy	17/39 (43.6%)	6/10 (60%)	0.5
Underlying condition (%)			
Without chronic disease	10/39 (25.6%)	5/10 (50%)	0.08
Diabetes mellitus	16/39 (41%)	3/10 (30%)	0.08
CIHD^c^	12/39 (30.7%)	2/10 (20%)	0.16
Hypertension	1/39 (2.5%)	0	NA
Chronic therapy (%)			
Without chronic therapy	10/39 (25.6%)	5/10 (50%)	0.04
Statins	13/39 (33.3%)	3/10 (30%)	0.48
ACE^d^	16/39 (41%)	2/10 (20%)	0.03
Postoperative complications			
Intraperitoneal leak (*n*, %) (documented)	27/39 (69.2%)	9/10 (90%)	0.01
Leak location (n, %)			
Gastroesophageal junction	8/39 (34.8%)	8/10 (80%)	0.02
Gastrointestinal anastomosis	7/39 (30.4%)	1/10 (10%)	0.01
Duodenum/small bowel	8/39 (34.8%)	1/10 (10%)	0.02
Intra-abdominal abscesses (*n*, %)	16/39 (41%)	9/10 (90%)	0.006
Presence of pleural effusion (*n*, %)	4/39 (10.3%)	4/10 (40%)	0.04
Clinical outcome endpoints			
APACHE 24^e^ (units, mean ± SD)	24.51 ± 6.06	24.2 ± 5.37	0.67
TISS score 24^e^ (units, mean ± SD)	22.48 ± 6.03	22.2 ± 5.37	0.72
ICU length of stay (day, mean ± SD)	11.6 ± 1.5	13.9 ± 1.4	0.65
Hospital length of stay (day, mean ± SD)	34.84 ± 2.6	25.2 ± 1.5	0.11
ICU mortality (%)	6/39 (15.4%)	5/10 (50%)	0.03

^*∗*^ *p* < 0.05 was found to be statistically significant. ^a^Invasive fungal (*Candida*) complications: *Candida peritonitis*, candidemia, and *Candida empyema*. ^b^Gastrointestinal tract; ^c^CIHD: chronic ischemic heart disease; ^d^ACE: angiotensin-converting enzyme. ^e^Within 24 hours of ICU admission.

**Table 3:** Microbiological data (from intraabdominal fluid, blood, and pleural effusions) and other laboratory parameters of the Group 2 patients during their ICU stay.

	Nonfungal (*n* = 39)	Invasive fungal^a^ (*n* = 10)	*p* value^*∗*^
*Intraabdominal positive cultures (%):*			
No organisms	22/39 (56.4%)	5/10 (50%)	0.59
*E. coli *	10/39 (25.6%)	2/10 (20%)	0.61
*Klebsiella* spp.	1/39 (2.6%)	0	NA
*Enterococcus* spp.	1/39 (2.6%)	0	NA
*Staph. aureus*	1/39 (2.6%)	0	NA
*Staph. coagulase* negative	1/39 (2.6%)	0	NA
*Streptococcusconstellatus* ^b^	1/39 (2.6%)	1/10 (10%)	0.17
*Pseudomonas* spp.	1/39 (2.6%)	0	NA
*Pleural effusion positive cultures (%)*			
No organisms	35/39 (89.7%)	9/10 (90%)	0.8
*Streptococcusconstellatus* ^b^	2/39 (5.1%)	1/10 (10%)	0.75
*Staph. coagulase* negative	1/39 (2.6%)	0	NA
*Pseudomonas* spp.	1/39 (2.6%)	0	NA
*Blood cultures (%):*			
No organisms	21/39 (53.8%)	5/10 (50%)	0.35
*E. coli*	1/39 (2.6%)	0	NA
*Klebsiella* spp.	8/39 (20.5%)	0	NA
*Staph. coagulase* negative	4/39 (10.3%)	4/10 (40%)	0.23
*Streptococcus constellatus*	2/39 (5.1%)	1/10 (10%)	0.3
*Pseudomonas* spp.	3/39 (7.6%)	0	NA
*Laboratory data* ^b^ *:*			
WBC (cells/mcq, mean ± SD)	16410.7 ± 1421.4	17800 ± 1389.4	0.41
Neutrophil (%)	85.8 ± 8.4	83 ± 11.1	0.27
Creatinine (mg/dl)	0.94 ± 0.58	1.6 ± 0.2	0.01
Phosphorus (mmol/L)	3.78 ± 1.4	4.06 ± 1.44	0.75
pH arterial blood	7.31 ± 0.1	7.32 ± 0.12	0.24
Lactate arterial blood (mmol)	1.96 ± 1.8	2.67 ± 0.7	0.11

^*∗*^ *p* < 0.05 was found to be statistically significant. ^a^Invasive fungal (*Candida*) complications: *Candida peritonitis*, candidemia, and *Candida empyema*. ^b^All laboratory data presented are those recorded on admission to the ICU.

**Table 4:** Multivariate logistic regression analysis of risk factors for postoperative secondary peritonitis after oncologic upper GIT surgery.

	OR	95% CI	*p* value
Age	1.1	1.07–1.29	0.001
Diabetes mellitus (type II)^a^	4.3	1.56–13.1	0.001
Total parenteral nutrition	1.1	1.0–1.9	0.04

^a^Underlying medical conditions.

**Table 5:** Multivariate logistic regression analysis of risk factors for invasive fungal infection after oncologic upper GIT surgery.

	OR	95% CI	*p* value
Age	1.2	1.07–1.29	0.04
Gastroesophageal leak^a^	2.66	1.16–5.26	0.02

^a^Documented intraperitoneal leak location during first recurrent surgical procedure.
